# New recorded diatoms in Holocene sediment cores from the Gonggeom-ji Wetland in Korea

**DOI:** 10.1186/s42649-023-00086-5

**Published:** 2023-01-20

**Authors:** Daeryul Kwon, Mirye Park, Hoil Lee, Jin-Young Lee, Sang Deuk Lee

**Affiliations:** 1 Protist Research Team, Microbial Research Department, Nakdonggang National Institute of Biological Resources, 137 Donam 2-Gil, Sangju-Si, 37182 South Korea; 2 Active Tectonics Research Center, Korea Institute of Geoscience and Mineral Resources, Geologic Hazards Division, 124 Gwanhak-Ro, Yuseong-Gu, Daejeon, 34132 South Korea; 3 Climate Change Response Division, Korea, Institute of Geoscience and Mineral Resources, Quaternary Environment Research Center, 124 Gwanhak-Ro, Yuseong-Gu, Daejeon, 34132 South Korea

**Keywords:** Fossil diatoms, Microscopic analyses, Morphology, Novel species, Paleontology

## Abstract

The Gonggeom-ji reservoir is an agricultural one built for rice farming during the Proto-Three Kingdoms period and was designated as Gyeongsangbuk-do monument No. 121 because of its high historical value. The Nakdonggang National Institute of Biological Resources has been conducting paleontological and paleoenvironmental studies on major wetlands from Korea since 2016, as well as diatom, geological, and depth distribution analyses on the sedimentary soil of Gonggeom-ji. This study summarized the description and ecological characteristics of six newly recorded diatoms (*Gomphonema lacusrankala, Pinnularia diandae, P. gibba* var. *hyaline, P. lacunarum, Sellaphora labda* var. *nipponica, Stauroneis angustilancea*) found in samples collected through drilling in Gonggeom-ji in 2019.

## Introduction

Wetlands are areas where land and aquatic habitats are covered with water and these primary factors control the water environment and sustain the life of animals and plants (Moon 2006; Ramachandra et al. 2005). Wetlands are also recognized as having rich nutrient content and is the most productive ecosystem on Earth, providing ecological, social, and economic benefits (Kwon and Choi 2009; Mulamootti et al., 1996; Ramachandra et al. 2005; Ramsar, 2013; Woodward and Wui 2001). Since the implementation of the Convention on Biological Diversity, the importance of biodiversity has emerged, and many efforts have been made to secure and sustainably use it in each country (Kim et al. 2017; Song and Park 2013). Recently, the importance of wetlands is becoming increasingly recognized and many studies are being conducted on them. Although studies on the classification of wetlands, such as stream, urban, and mountain wetlands, and national wetland protection areas are continuously conducted, those in rural areas are relatively scarce (Ji 2008; Park et al. 2006; Son et al. 2012). The Gonggeom-ji reservoir is an agricultural reservoir built for rice farming during the Proto-Three Kingdoms period and was designated as Gyeongsangbuk-do monument No. 121 due to its high historical value. It is one of the oldest artificial reservoirs in the Joseon Dynasty period, along with Uirim-ji (Jecheon) and Byeokgol-je (Gimje) reservoirs (Daegu Regional Environmental Office, 2013; Lim et al. 2011). In addition, due to the emergence of legally protected species and abundant biodiversity, 0.264 km^2^ around Gonggeom-ji reservoir was designated as the first wetland protection area in Korea based on the Wetland Conservation Act (Daegu Regional Environmental Office, 2013). Since Gonggeom-ji has a very high historical value, many related studies are also being conducted, such as research on understanding the construction period and the land and aquatic environment around Gonggeom-ji ( Kim et al. 2017; Lim et al. 2011). The Nakdonggang National Institute of Biological Resources conducted diatom, geological, and depth distribution analyses on the sedimentary soil of Gonggeom-ji in 2019 and 2020 and recently revealed the size of the reservoir (Lee et al. 2020a, b). Diatoms, which played an important role in determining the size of Gonggeom-ji, are single-celled eukaryotic groups of microalgae called yellow or golden algae (Falciatore and Bowler 2002; Mann et al. 2010). Diatoms can be found free-floating or attached to surfaces in oceans, freshwater, and brackish waters, and have chlorophyll, which is important for their role as primary producers in water systems (Evans et al. 2007; Round et al. 1990; Valentin et al. 2019). In particular, diatoms are the most significant microalgae that can represent the past environment because they can easily remain fossilized in sedimentary layers due to their solid siliceous cell walls (Gell et al. 2005; Lotter 1998), and has the advantage of allowing for the ecology of fossil species to be studied (Pike et al. 2008). In Korea, fossil diatom research began in the early 1970s and still continues today. However, diatom research in the freshwater wetland sedimentary layer remains lacking (Lee 1996; Lee et al. 2020a, b), and most studies on domestic wetlands and sedimentary layers have focused on geology and pollen (Yi et al. 2005; Yoon et al. 2008). Our study aimed to summarize the description and ecological characteristics of newly recorded diatoms (*Gomphonema lacusrankala, Pinnularia diandae, P. gibba* var. *hyaline, P. lacunarum, Sellaphora labda* var. *nipponica, Stauroneis angustilancea*) found in samples collected through drilling in Gonggeom-ji in 2019.

## Materials and Methods

### Sediment drilling and age dating

**Fig. 1:**
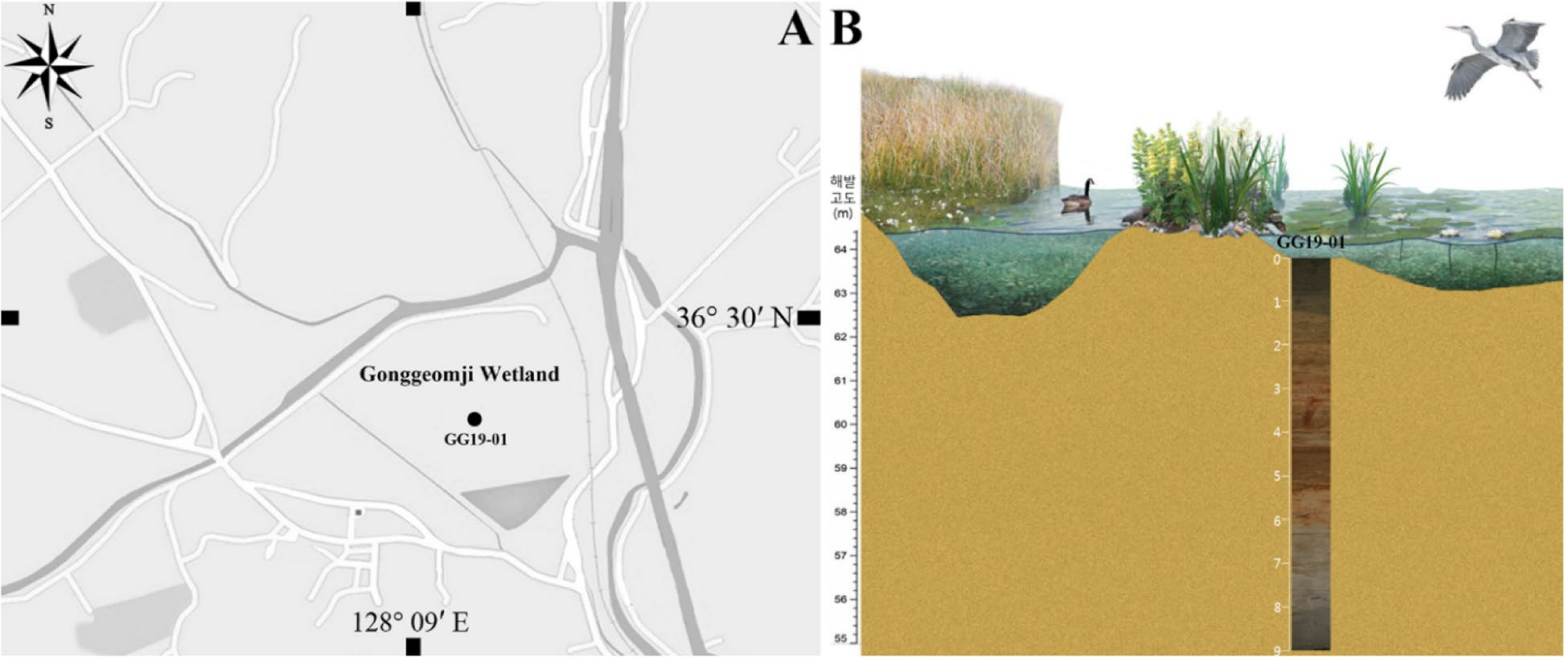
Map of the study area (**A**) and drilling location (**B**)

Drilling was carried out using a peat sampler (52.4 mm diameter; Eijkelkamp, Netherlands) on a small barge in the center of Gonggeom-ji, referred to as sampling site GG19-01 (Fig. 1). The recovered core sediments were vacuum-packed in waterproof plastic bags and subsampling performed after they were transported to the laboratory. Age dating was performed using an accelerator mass spectrometer at the Korean Institute of Geoscience and Mineral Resources (South Korea) using plant materials. Estimated ages were corrected using the OxCal statistical analysis program (http://c14.arch.ox.ac.uk).

### Diatom sample preparation and identification

A 1 g sample of dried sediment was placed in a 50 mL beaker containing 35.5% hydrogen peroxide (Junsei Chemical Co., Ltd., Japan) and 15 mL distilled water and heated on a hot plate (SMHS-6; Daihan Scientific Co. Ltd., South Korea) for 3–4 h. When the sample reaction occurred and sediments were stationary, the dilution and re-station process was repeated three times by adding sufficient distilled water. Thereafter, the supernatant of hydrogen peroxide and distilled water in the beaker was removed using a gradient method. The pretreated sample was stored in a corning tube. To analyze the shape of diatoms, coverslips were diluted with 50 μL pretreated sample and distilled water on a slide warmer (XH-2001, Premiere, USA), which was then heated and dried. When drying was completed, ethanol was added to the pleurax (Mountmedia, Wako, Japan), and the concentration was adjusted. Thereafter, 1–2 drops were added to the dried sample, covered with a glass slide, and pressed to allow the mounting agent to penetrate. Completely hardened slide samples were preciese identified using a light microscope (Nikon, Eclipse Ni-U) equipped with Nomarski differential interference contrast optics. Micrographs were taken using a digital camera (DS-Ri2; Nikon, Japan). The remaining peroxide-cleaned samples were filtered using 2.0 μm nuclepore polycarbonate membrane filters (Whatman plc, UK). The membranes were placed on stubs and coated with gold–palladium for analysis using a field-emission scanning electron microscope (MIRA 3; TESCAN, Czech Republic). Dimensions of the diatoms were measured using image v1.32 software (Schneider et al. 2012). Taxonomic nomenclature was based on recent taxonomic information (AlgaeBase, https://www.algaebase.org).

## Results

### *Gomphonema lacus-rankala Gandhi*^1958^ (Figs. 3A and B)

**Original description**: HP Gandhi, Freshwater diatoms from Kolhapur and its immediate environs. J. Bombay Nat. Hist. Soc. 55, 493–511 (1958).

**General environment:** Freshwater species.

**Fig. 2:**
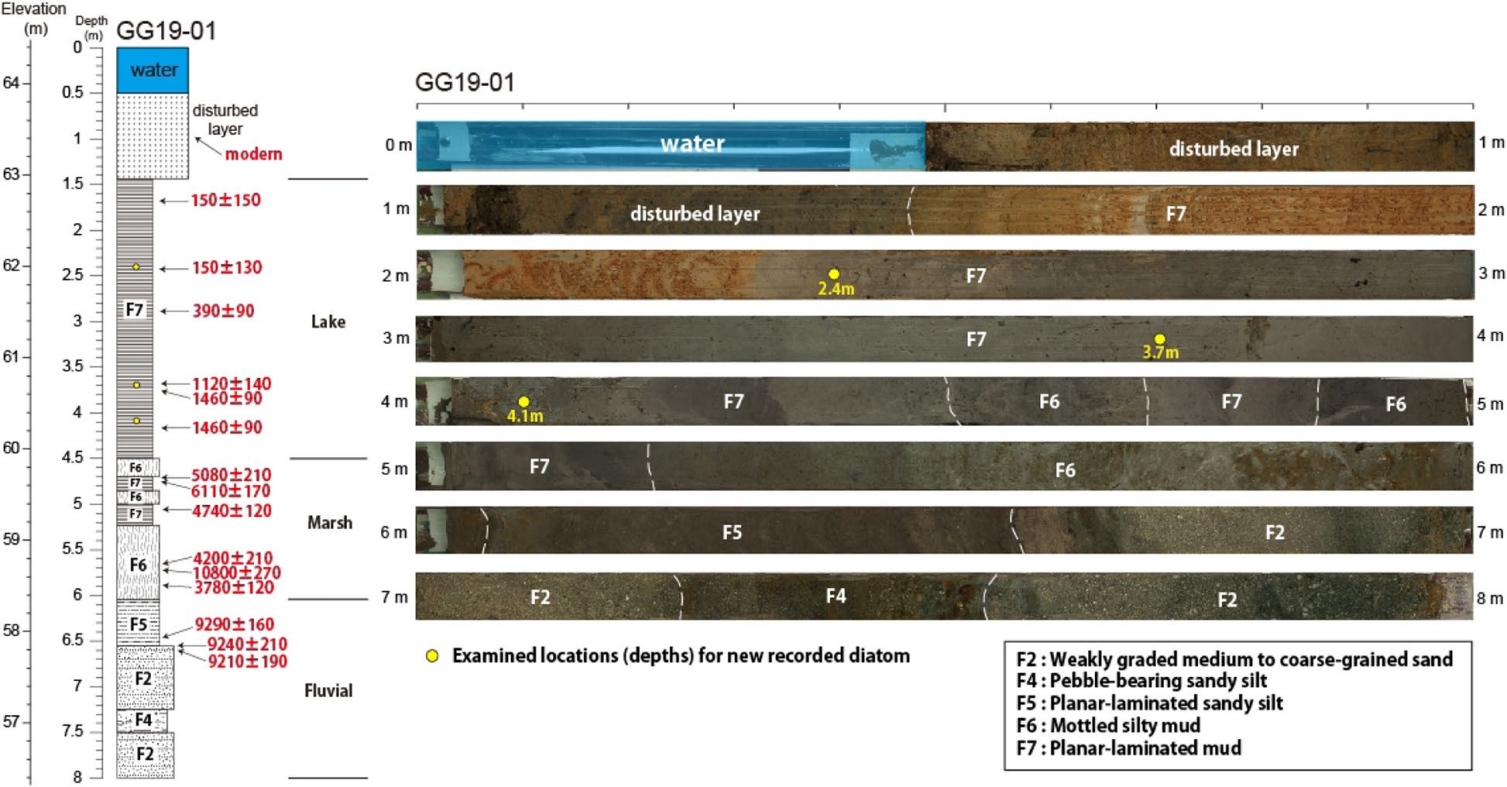
Stratigraphic section, age dating, and photographs of the sediment core at sampling site GG19-01

**Sampling depth**: 2.4 m, GG19-01 (Fig. 2, Table 1).

**Table 1:** Accelerator mass spectrometer ^14^C dating and calibrated ages for core GG19-01 sediment samples

Depth (m)	Elevation (m)	^14^C yr BP (± 1σ)	Cal. yr BP (± 2σ)	Laboratory code	Dating material
0.98	63.41	150 ± 40	Modern	KGM-OWd190268	Plant fragment
1.68	62.71	170 ± 40	300 ± 150	KGM-OWd190270	Plant fragment
2.43	61.96	110 ± 30	280 ± 140	KGM-OWd190271	Plant fragment
2.89	61.5	330 ± 30	500 ± 100	KGM-OWd190272	Plant fragment
3.68	60.71	1190 ± 40	1260 ± 140	KGM-OWd190274	Plant fragment
3.75	60.64	1570 ± 40	1550 ± 90	KGM-OWd190275	Plant fragment
4.17	60.22	1560 ± 40	1550 ± 100	KGM-OWd190276	Plant fragment
4.71	59.68	4420 ± 50	5290 ± 210	KGM-OWd190277	Plant fragment
4.75	59.64	5310 ± 70	6280 ± 180	KGM-OWd190278	Plant fragment
5.06	59.33	4210 ± 40	4850 ± 130	KGM-OWd190280	Plant fragment
5.66	58.73	3780 ± 60	4410 ± 220	KGM-OWd190281	Plant fragment
5.72	58.67	9440 ± 50	11,060 ± 270	KGM-OWd190282	Plant fragment
5.89	58.5	3520 ± 40	3900 ± 120	KGM-OWd190283	Plant fragment
6.46	57.93	8300 ± 50	9470 ± 170	KGM-OWd190284	Plant fragment
6.55	57.84	8280 ± 60	9460 ± 210	KGM-OWd190285	Plant fragment
6.6	57.79	8200 ± 60	9400 ± 200	KGM-OWd190286	Plant fragment

**Fig. 3:**
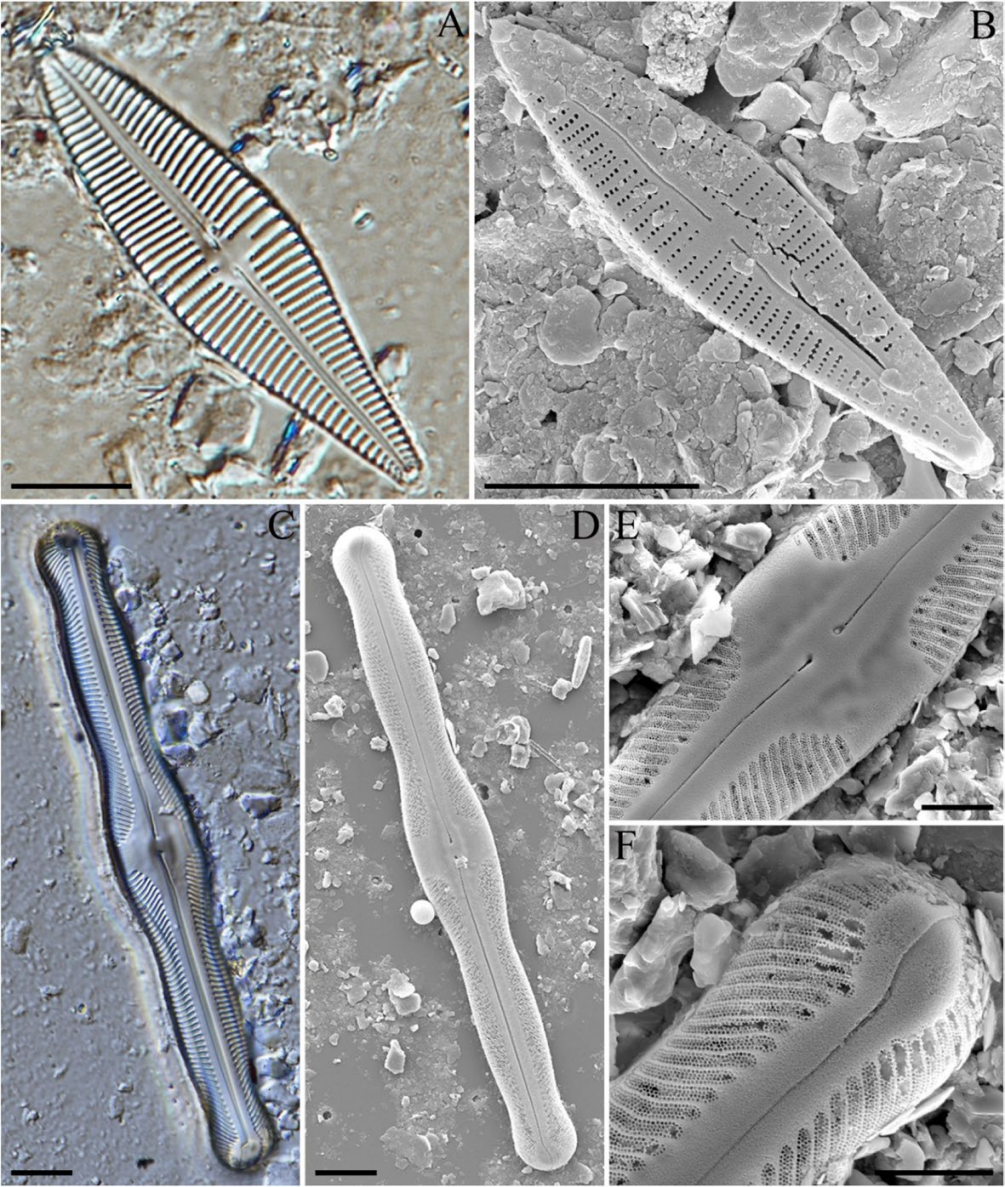
Light (**A**, **C**) and field emission scanning electron (**B**, **D**–**F**) photomicrographs of diatoms collected from the sediment core at sampling site GG19-01. (**A**, **B**) *Gomphonema lacusrankala*. (**C**–**F**) *Pinnularia diandae.* Scale bars: A–D = 10 μm; E, F = 5 μm

**Description:** Broad to elliptical lanceolate valves with protracted and slightly rounded apices. Length range: 69–96 μm, width range: 16–18 μm. The axial area was narrow, forming a narrow, slightly asymmetrical central area. Striae were punctate, slightly radiate at the center, strongly radiate towards the ends, and 9–12 in number with a 10 μm length. One stigma was present at the end of the central stria. Raphe undulates and were lateral. External proximal raphe ends were deleted, and the internal ends recurved. The external distal raphe ends were curved onto the mantle (Fig. 3A). SEM showed that the valve exterior had the characteristic protracted head pole, distinct central area with dilated proximal raphe ends, and round stigma opening. At the head pole, the raphe was deflected to one side and extended to the valve mantle (Fig. 3B).

### *Pinnularia diandae Metzeltin* et Lange-Bertalot 2007 (Figs. 3C–F)

**Original description**: D Metzeltin, Tropical diatoms in South America II: Special remarks on biogeographic disjunction (A. R. G. Gantner, Ruggell, 2007).

**General Environment:** Freshwater species.

**Sampling Depth**: 4.1 m, GG19-01 (Fig. 2, Table 1).

**Description:** Valves generally had similar shapes, but the tips were wider and attached to the head rather than extending beyond. They were different in their dimensions for length (137–142 μm) and had a width of approximately 20 μm. Raphes with central pores were located between each other. Raphes appeared as straight lines, but can be tilted towards the central area and slightly on the apex axis. Striae displayed the same pattern of radial, subparallel, and convergent positions, but were coarser. There were only eight striae with a length of 10 μm (Fig. 3C). SEM showed that the areola was symmetrical with respect to the raphe, and these pores showed irregular arrangements. Overall, they showed a thick, tube-like striae shape (Fig. 3D). The areola was connected to the side of the valve. The striae are symmetrical left and right at a predetermined interval based on the raphe and are inclined towards the center of the raphe (Figs. 3E and F).

### *Pinnularia gibba var. hyalina Hustedt*^1942^ (Figs. 4A–G)

**Fig. 4:**
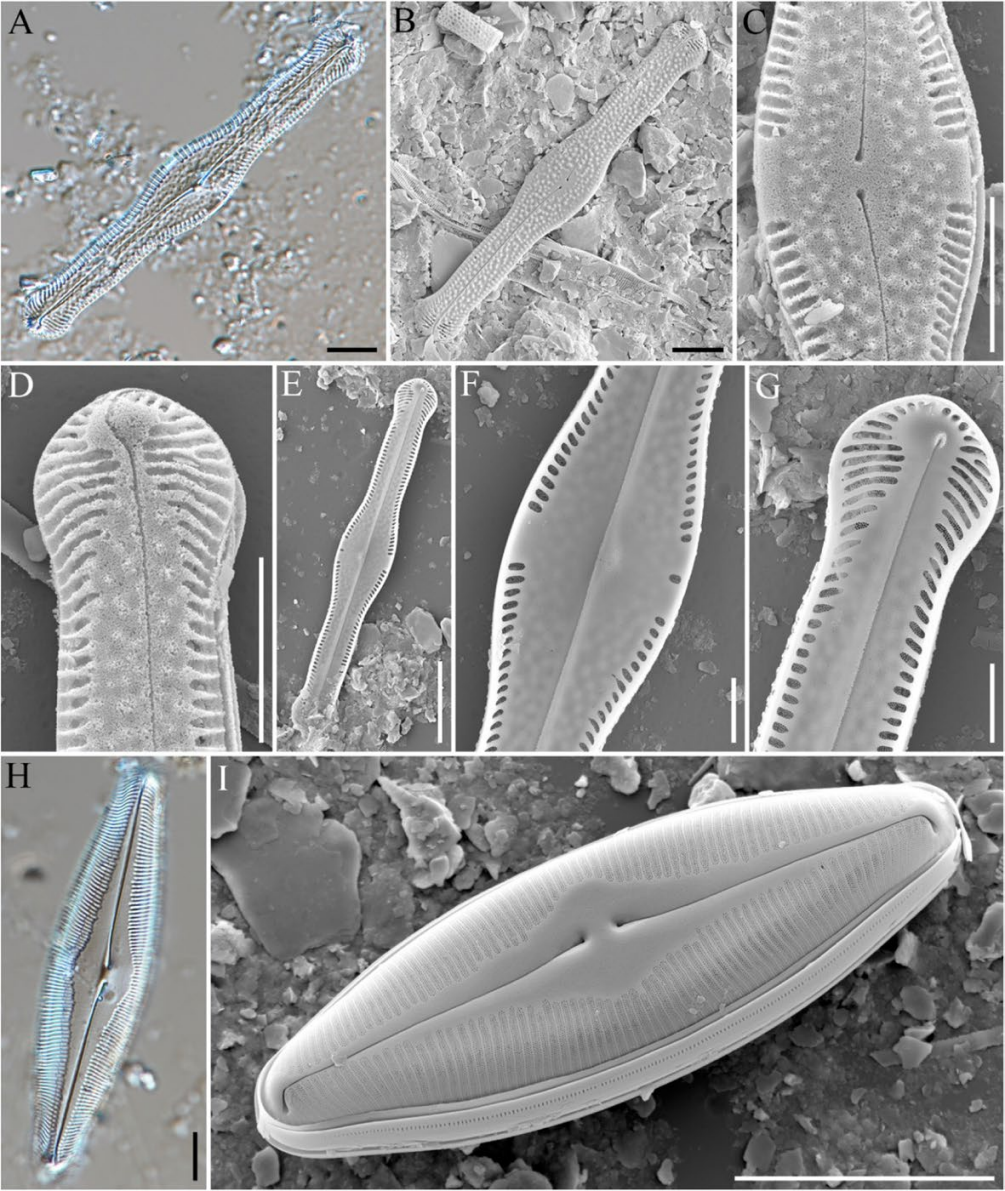
Light (**A**, **H**) and field emission scanning electron (**B**–**G**, **I**) photomicrographs of diatoms collected from the sediment core at sampling site GG19-01. (**A**–**G**) *Pinnularia gibba* var. *hyaline*. (**H**, **I**) *P. lacunarum*. Scale bars: A–D, H = 10 μm; E, I = 20 μm; F, G = 5 μm

**Synonym:** *Pinnularia hyaline* Hustedt 1934.

**Original description**: F Hustedt, Süßwasser-Diatomeen des indomalayischen Archipels und der Hawaii-Inseln. Nach dem Material der Wallacea-Expedition. Int. Rev. ges. Hydrobiol. und Hydrogr. 42 (1–3), 1–252 (1942).

**General Environment:** Freshwater species.

**Sampling Depth**: 2.4 m, GG19-01 (Fig. 2, Table 1).

**Description:** The valve was linear, central undulation broader, ends broadly capitate, somewhat flattened, and as wide as the valve body. The length range was 90–105 μm, and the width range was 12–13 μm. Axial area: broad, parallel sides forming a wide, slightly asymmetrical central area. Striae were radiate in the median portion of the valve, somewhat convergent or parallel towards the ends, and 12–14 in number that were 10 μm long. The raphe filiform and terminal fissures were distinct and semicircular (Fig. 4A). SEM showed traces of swelling on the axial area and were widely spread, except for some of the central area (Figs. 4B–D). The axial plate was connected with an extended axial costa that was in the form of a thin line (Figs. 4E and F). Striae were hollowed out, and those around the polar raphe symmetrical in a narrow arrangement, but the gap was wider as they reached the central area (Figs. 4C, D, F, and G).

### *Pinnularia lacunarum *Hustedt 1942 (Fig. 4H, I)

**Original description:** F Hustedt, Süßwasser-Diatomeen des indomalayischen Archipels und der Hawaii-Inseln. Nach dem Material der Wallacea-Expedition. Int. Rev. ges. Hydrobiol. Hydrogr. 42(1/3): 1–252 (1942).

**General Environment:** Freshwater species.

**Sampling Depth**: 4.1 m, GG19-01 (Fig. 2, Table 1).

**Description:** Valves were linear to rhombic, lanceolate, and ends broadly rounded. Length range: 75–110 µm, width range: 11–24 μm. The axial area was linear or tapered towards the ends and half the breadth of the valve. The central area was large, roundish to rhombic, a third to one-half the breadth of the valve, and commonly asymmetrical. Striae were approximately 9–10 in number with a length of 10 μm, radiate in the middle, convergent towards the ends, and longitudinal bands absent. The raphe filiform was terminally distinct and semicircular (Fig. 4H). SEM clearly showed that it was radial based on the central area. The axial area became wider as it went to the central area, and the central raphe ends were hollowed. The raphe connected to the central area continued to the polar nodule and were bent greatly at the apex. The striae was composed of a porous areola and had a tube-like shape (Fig. 4I).

### *Sellaphora lambda var. nipponica *(Skvortsov) T. Ohtsuka 2002 (Figs. 5A and B)

**Basionym:** *Navicula lambda* var. *nipponica* Skvortsov 1936.

**Original description**: T Ohtsuka, A Tuji, Lectotypification of some pennate diatoms described by Skvortzow in 1936 from Lake Biwa. Phycological Res. 50 (4), 243–250 (2002).

**General Environment:** Freshwater species.

**Sampling Depth**: 3.7 m, GG19-01 (Fig. 2, Table 1).

**Fig. 5:**
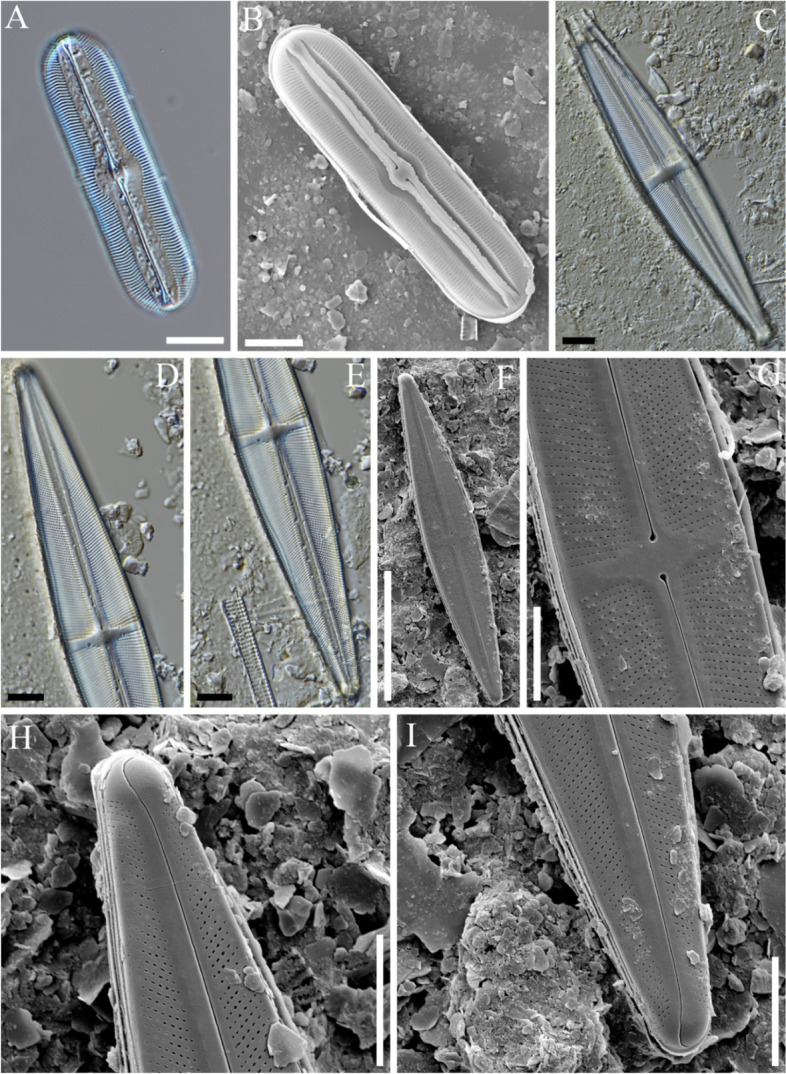
Light (**A**, **C**, **D**, **E**) and field emission scanning electron (**B**. **F**–**I**) photomicrographs of diatoms collected from the sediment core at sampling site GG19-01. (**A**, **B**) *Sellaphora labda* var. *nipponica*. (**C**–**I**) *Stauroneis angustilancea*. Scale bars: A–E, G–I = 10 μm; F = 50 μm

**Description:** Valves were linear with parallel margins and broad with obtuse ends. Length range: 44–68 µm, width range: 10–14 µm. The axial area was broad. The central area was wider than the axial area, had a distinct central nodule, and was slightly asymmetrical. Densely spaced striae were porous, slightly radiate at the center, and strongly radiate towards the ends. The raphe stretched straight from the apex to the terminal end and the wide sternum was clearly visible (Fig. 5A). SEM showed that the sternum was hollowed out, the area surrounding the raphe protruded, and central raphe area became very thick. Areolae were lined up in a porous row, radially symmetrical and radial as they approached the apex axial area (Fig. 5B).

### *Stauroneis angustilancea *Lange-Bert & Metzeltin 2004 (Figs. *5*C–I)

**Original description**: M Werum, H Lange-Bertalot, E Reichardt, Diatoms in springs from Central Europe and elsewhere under the influence of hydrogeology and anthropogenic impacts (A. R. G. Gantner Verlag K. G., Königstein, 2004).

**General Environment:** Freshwater species.

**Sampling Depth**: 3.7 m, GG19-01 (Fig. 2, Table 1).

**Description:** The valves were lanceolate with gradually attenuated, weakly protracted, narrow, and subrostrate apices. Length range: 79–125 μm, width range: 15–21 μm. The central area was narrow, linear, and somewhat flaring near the axial area. The central area was a rectangular structure that expanded slightly near valve margins. The raphe branches were lateral, and the proximal raphe ends weakly deflected to one side and slightly inflated. Terminal raphe fissures were shaped like question marks and open towards the secondary side. Striae were radiate throughout and more strongly towards the valve apices. Areolae were relatively coarse and numbered 15–20 at a length of 10 µm (Figs. 5C–E). SEM showed lineolate areolae and striae were densely spaced. Striae were aligned in a row and the radial shape strongly formed from the central area to the polar nodule. The stauros was quite slanted, and the angle of the valve margin was nearly vertical in the central area. The gap between the stauros widened as it approached the valve margin. In the polar nodule, no striae were found, and the raphe was connected to the end (Figs. 5F–I).

## Discussion

### Taxonomic and distributional characteristics

Little is known about the morphological and distributional characteristics of these six unrecorded species. In this study, the ecological and morphological characteristics of six diatoms at the genus level are discussed. The genus, *Gomphonema*, comprises both cosmopolitan and endemic species that exhibit a wide range of ecological preferences (Ehrenberg 1854; Karthick and Kociolek 2012). The National List of Species of Korea (2021) has recorded 69 species of *Gomphonema*. *Pinnularia* is a cosmopolitan genus distributed worldwide (Ciniglia et al. 2007), of which The National List of Species of Korea (2021) has recorded 146 species. Over 1500 *Pinnularia* spp. have been reported worldwide, with many species being found in oligotrophic, low-electrolyte-content freshwater (Krammer 2000; Metzeltin and Lange-Bertalot 1998). Generally, *Pinnularia* found in extreme environments is also present in freshwater and terrestrial habitats, and their occurrence in acid-thermal sites may either be connected to simple tolerance to a wide pH range, the activation of specific metabolic pathways, or selection (Ciniglia et al. 2007. Most diatoms belonging to Naviculaceae are characterized by the fact that the line pattern running horizontally on the lid is connected by a simple areola, whereas that of *Pinnularia* is connected by a long chamber. About 6000 species of Naviculales have been reported worldwide, but taxonomic recombination has recently included several genera, including *Anulemastus*, *Craticula*, *Fallacia*, *Placoneis*, and *Sellaphora* (AlgaeBase, https://www.algaebase.org). The reason for this complex taxonomic recombination is inferred from the classification of many species into a single genus and bearing in mind only morphological similarities in the absence of various taxonomic considerations in the past (Wehr and Sheath 2003). There are approximately 247 species of the genus, *Sellaphora*, worldwide (AlgaeBase, https://www.algaebase.org), and seven species have been reported in Korea (National List of Species of Korea, 2021). The *Stauroneis* genus is widely distributed in fens, ponds, and small lakes.

## Paleoenvironmental change

In GG19-01 core sediment, *G. lacusrankala* and *P. gibba* var. *hyaline* occurred at the drilling depth of 2.4 m, *S. lambda* var. *nipponica* and *Stauroneis angustilancea* at 3.7 m, and *P. diandae* and *P. lacunarum* at 4.1 m. These depths belong to a lake environment characterized as planar-laminated clay (sedimentary facies F7; Fig. 2). The ages of these three depths were estimated to be 1460, 1120, and 150 cal. yr BP from the lower to the upper part. They showed a relatively large time interval between 3.7 m and 2.4 m in the same lake system (Fig. 2 and Table 1) (Lee et al. 2018; Yi et al. 2020). Based on the age dating results, it appears that the fluvial environment was dominant until approximately 3700 cal. yr BP. Then, it changed to a marsh environment before forming a lake at approximately 1400 cal. years BP (Fig. 2). This coincides with the formation of the Gonggeom-ji reservoir at 1350 yr BP, suggesting that it became a lake because of the construction of the embankment (Lee et al. 2018). Although it is difficult to estimate the exact time of environmental change because age dating results show high variability until a depth of 4.5 m before the lake environment, it seems that the lake had been maintained stably after the construction of the embankment. It is also supported that the location of this core sediment is inside the reconstructed Gonggeom-ji reservoir, showing a deeper depth than the drilling results of a previous study (Lee et al. 2018). Thus, the new record of six species appeared to be inhabited in a stable lake environment.

## Conclusions

Six diatom species were new records for Korea: *Gomphonema lacusrankala*, *Pinnularia diandae*, *Pinnularia gibba* var. *hyaline*, *Pinnularia lacunarum*, *Sellaphora lambda* var. *nipponica*, *Stauroneis angustilancea*. The morphological characteristics of the six newly recorded species were described and illustrated based on the high-quality Light Microscopy and field-emission scanning electron microscopic photographs. We discussed the ecological and morphological characteristics of six diatoms at the genus level, and the Paleoenvironmental change based on the accuring depth and agae dating results.
